# Adult Inpatients’ Perceptions of Their Fall Risk: A Scoping Review

**DOI:** 10.3390/healthcare10060995

**Published:** 2022-05-27

**Authors:** Elissa Dabkowski, Simon Cooper, Jhodie R. Duncan, Karen Missen

**Affiliations:** 1 Institute of Health and Wellbeing, Federation University Australia, Gippsland, VIC 3842, Australia; s.cooper@federation.edu.au (S.C.); karen.missen@federation.edu.au (K.M.); 2 Research Unit, Latrobe Regional Hospital, Traralgon, VIC 3844, Australia; jduncan@lrh.com.au

**Keywords:** falls, patient, perception, hospital, fall prevention, falls risk assessment

## Abstract

Patient falls in hospitals continue to be a global concern due to the poor health outcomes and costs that can occur. A large number of falls in hospitals are unwitnessed and mostly occur due to patient behaviours and not seeking assistance. Understanding these patient behaviours may help to direct fall prevention strategies, with evidence suggesting the need to integrate patients’ perspectives into fall management. The aim of this scoping review was to explore the extent of the literature about patients’ perceptions and experiences of their fall risk in hospital and/or of falling in hospital. This review was conducted using a five-stage methodological framework recommended by Arksey and O’Malley. A total of nine databases were searched using key search terms such as “fall*”, “perception” and “hospital.” International peer-reviewed and grey literature were searched between the years 2011 and 2021. A total of 41 articles, ranging in study design, met the inclusion criteria. After reporting on the article demographics and fall perception constructs and measures, the qualitative and quantitative findings were organised into five domains: Fall Risk Perception Measures, Patients’ Perceptions of Fall Risk, Patients’ Perceptions of Falling in Hospital, Patients’ Fear of Falling and Barriers to Fall Prevention in Hospital. Approximately two-thirds of study participants did not accurately identify their fall risk compared to that defined by a health professional. This demonstrates the importance of partnering with patients and obtaining their insights on their perceived fall risk, as this may help to inform fall management and care. This review identified further areas for research that may help to inform fall prevention in a hospital setting, including the need for further research into fall risk perception measures.

## 1. Introduction

Patient safety in healthcare settings continues to be recognised as a global health priority. Current evidence shows that up to 83% of harm to patients is avoidable, producing additional costs of up to 15% of hospital expenditure in high-income countries [1]. Falls in hospitals constitute one of the greatest sources of patient harm on a global scale, with up to 80% of falls occurring in low- to middle-income countries [1]. Approximately 700,000 to 1 million patient falls occur in hospitals in the United States of America alone, contributing to 250,000 injuries and up to 11,000 deaths [2]. Patient falls continue to be a high priority for healthcare organisations due to the detrimental physical, psychological, social and financial consequences that can occur.

## 2. Background

Despite decades of research, there is a lack of robust evidence relating to the efficacy of fall interventions in hospitals, including exercise regimes, medication reviews, bed alarms, patient education or assistive technology [3]. A worldwide taskforce has been established to update fall prevention and clinical management guidelines [4] with the intent to include patients/clients as stakeholders and to incorporate their perspectives of fall prevention and management. Previously, there has been minimal collaboration with patients in the planning, development and evaluation of multifactorial fall prevention programs [5]. Partnering with consumers, including the health insights of patients and their families/carers, should be valued and integrated into all levels of healthcare [1].

Understanding patients’ views of their fall risk may inform fall prevention policies in hospital settings. For example, Heng, Slade [6] explored patients’ perceptions of fall prevention education in hospital, revealing that most inpatients did not recognise that they were at risk. This is consistent with other studies that also identified that a lack of insight resulted in a greater risk of falling and reduced adherence to fall prevention strategies [7,8]. Obtaining patients’ perspectives of their fall risk provides an opportunity for health professionals to explore these beliefs, potentially creating drivers for change [9].

With these issues in mind, the authors conducted a scoping review to investigate the literature concerning patients’ perceptions of their fall risk in hospital and/or of falling in hospital. A scoping review may be used for four purposes: to examine the extent of the literature on a given topic, to determine the usefulness in undertaking a full systematic review, to summarise and disseminate research findings and to identify gaps in the existing literature [10]. Scoping reviews can also provide clarification of key concepts in the literature and inform the manner in which research is conducted on a specific topic [11]. To the best of our knowledge, a scoping review has not been previously undertaken on this topic. A scoping review for this topic may help to direct future efforts for fall research related to the hospital environment.

## 3. Aims

The overarching aim of this review is to scope the literature pertaining to adult patients’ perceptions and experiences of falling in hospital. Specific research objectives for this review are (i) to gain an understanding of patients’ perceptions of their fall risk in a hospital setting, (ii) to determine if there is a need to undertake a systematic review on this topic and (iii) to identify gaps in the literature on patients’ perceptions of their fall risk in hospital. An a priori scoping review protocol was developed before commencement, as recommended by the Joanna Briggs Institute (JBI) for systematic scoping reviews (see Appendix A) [12].

## 4. Methods

### 4.1. Design

A scoping review is used to determine the breadth of research on a broad topic and map emerging concepts to identify research gaps [12]. Scoping reviews are useful when a body of literature has not been examined comprehensively or is heterogenous in nature, indicating that a systematic review may not be suitable [12]. A systematic review can be used to address research questions about the effectiveness, practicality or suitability of a treatment or type of clinical practice [11]. Given the broad nature of our research aim, a scoping review was the preferred typology. Therefore, the five-stage methodology devised by Arksey and O’Malley [10] was used to guide this review. The sixth stage of Arksey and O’Malley [10] is an optional stakeholder consultation exercise, which was omitted from this review. In addition, this review followed the evidence-based 22-item Scoping Review Checklist (SRC) developed by Cooper, Cant [13]. This 22-item Scoping Review Checklist (SRC) was rigorously developed through a series of expert consultation processes and can be used to guide the reporting and quality of scoping reviews [13].

### 4.2. Search Strategy

To develop the research question, the Population, Concept and Context (PCC) mnemonic was utilised as a guide to reflect a meaningful title and research question [14]. A literature search was conducted between July and September 2021. Before commencing, a search strategy was devised with an experienced university research librarian. An in-depth literature search was then conducted using the following databases to source both peer-reviewed studies and grey literature: CINAHL Complete, MEDLINE, APA PsycINFO, APA Psyc Articles, Web of Science, SCOPUS, Cochrane library, ProQuest and the search engine Google Scholar. These databases were searched using a Boolean search strategy, which included key concepts and their variations and truncated symbols (Table 1). Limitations were applied to the search results to include studies published within a ten-year time frame from the date of the search and articles in the English language. A scoping review is usually conducted without a date restriction; however, the authors collectively opted to use this ten-year time frame. The authors were specifically interested in the latest evidence about this topic, bearing in mind the extensive nature of fall research. The reference lists of identified papers were also searched to uncover additional studies. These search results were uploaded to a Covidence database (a software program for screening systematic reviews) [15] in order to facilitate research collaboration and the selection of papers in line with the inclusion and exclusion criteria.

### 4.3. Screening and Eligibility

The inclusion and exclusion criteria were developed by all four authors (ED, SC, JD and KM) to achieve general consensus about the eligibility criteria. The focus of the included studies was on perceptions or attitudes about falling or about their fall risk in hospital. The authors included a variety of constructs that conveyed perception, as shown in Table 1. Articles were included if participants were adults aged greater than 17 years and were hospital inpatients, including emergency departments. The exclusion criteria were studies that occurred in community or residential facilities/aged care and hospital outpatient clinics, including short-stay procedures. Studies were excluded if the focus of the paper was on the development of fall risk perception measures. The review considered all types of published papers that met the inclusion criteria.

**Figure 1:**
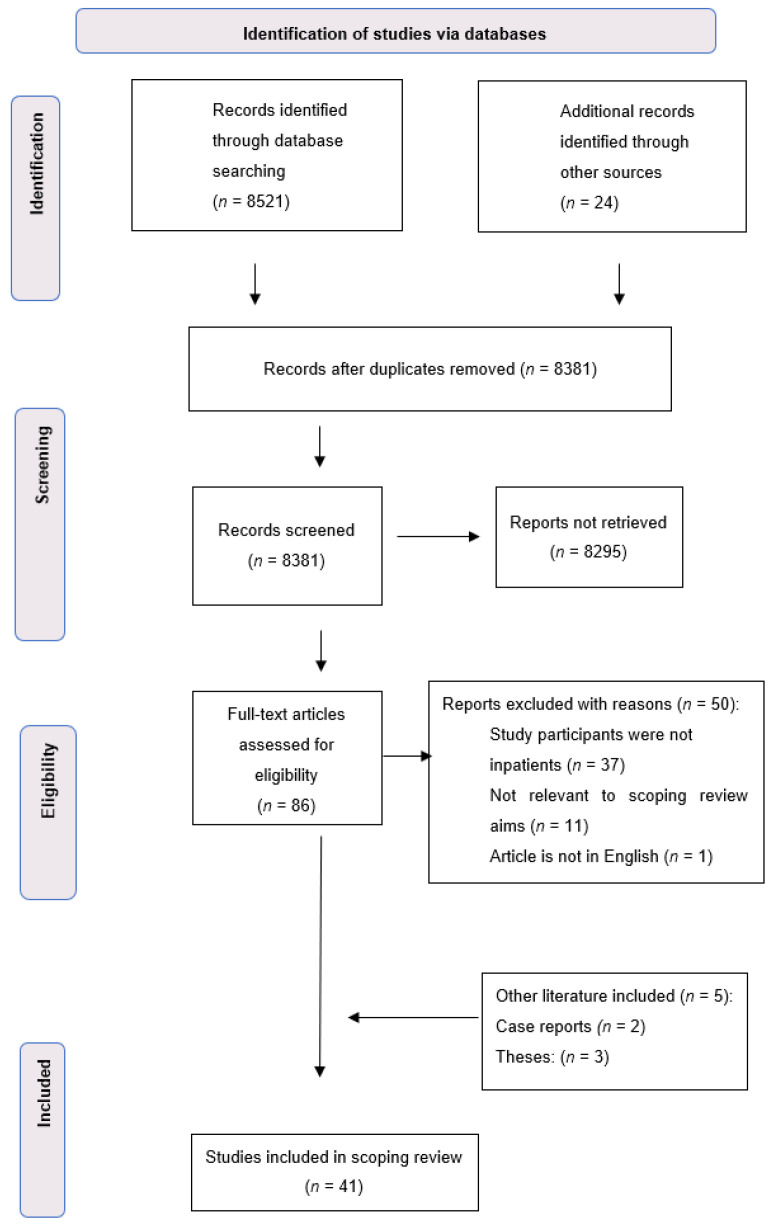
Flowchart of selection process.

After the search results were uploaded to Covidence [15] and duplications were removed, two authors (ED and SC) completed an independent title and abstract screen. In the event of uncertainty, a third author (KM) moderated the process until consensus was reached. The approved screened records were then obtained in full text by author ED and further evaluated by the research team to determine their relevance to the aims of the scoping review. All four authors approved the final list of articles for this scoping review, and a final check of selected papers was included to ensure that papers had not been retracted [13]. Figure 1 details the flow of the literature search process and study selection for this review.

### 4.4. Quality Appraisal

Quality appraisal is not always a required component of scoping reviews, given the potential to include grey literature [12]; however, this element is recommended by Cooper, Cant [13] to improve rigor. The quality appraisal process was completed by two authors independently, with a third author to moderate if a general consensus was not reached. An array of appraisal tools was utilised, depending on the individual study designs. Qualitative studies were appraised using the Critical Appraisal Skills Program (CASP) Qualitative Studies checklist [16], in which studies were scored on 10 items. Quantitative studies were evaluated on 12 items using the CASP Cohort Study Checklist [17] and the CASP Randomised Controlled Trial Checklist [18]. It is not always necessary to provide an overall score using CASP tools [16]; however, the authors opted to include overall scores given the summative scoring system of this quality appraisal process. The quality of quasi-experimental studies was appraised using the Joanna Briggs Institute (JBI) Critical Appraisal Checklist for Quasi-Experimental Studies [19]. Mixed methods studies were evaluated using the Mixed Methods Appraisal Tool (MMAT) [20]. Literature reviews were appraised using the six-item Scale for the Assessment of Narrative Review Articles (SANRA) [21]. The quality of case reports was assessed using the JBI Checklist for Case Reports [22], which consists of eight items. The doctoral dissertations and editorial column included in this scoping review were not subject to quality appraisal. The overall scores were included in a data summary table to rate the quality of evidence against validated quality appraisal tools.

### 4.5. Data Charting

A data charting form was developed based on the recommendations of Arksey and O’Malley [10] to map the key concepts and themes identified from the scoping review. After collaboration between all authors, it was decided that the following data were to be extracted verbatim: author, year and country, study aim, study design, study population, fall risk perception outcome measures and main findings. The data extraction was completed by the lead author, and all authors reviewed the extracted data to verify the final dataset. One corresponding author of a study was contacted via email on 12 August 2021 for additional information; however, they did not respond to our email. Consequently, we were unable to source further information about the fall risk perception measures reported in their study. In accordance with Cooper, Cant [13], a numerical analysis of the extent and nature of included studies was also reported.

### 4.6. Data Synthesis 

To provide a narrative account of the results, the authors familiarised themselves with the data and revisited the research objectives. The main qualitative and quantitative findings from each article were grouped into five domains: Fall Risk Perception Measures, Patients’ Perceptions of Fall Risk, Patients’ Perceptions of Falling in Hospital, Patients’ Fear of Falling and Barriers to Fall Prevention in Hospital. These domains were inductively developed from the findings of the review. The lead author collated the information into the five domains, providing a comparison between the relevant studies. All authors reviewed the domains and findings prior to summarising and reporting the results. Minor changes were made to the review protocol to incorporate the mapping of fall risk perception measures and identified barriers to fall prevention in hospitals. Results from a scoping review may be further refined towards the end of the review, as authors will have greater insight into the nature of the included studies [12].

## 5. Results

### 5.1. Article Characteristics

From the initial database search, a total of 8527 citations were identified, as shown in the PRISMA flowchart (Figure 1). Following a systematic process, 41 articles published between 2011 and 2021 were identified and included in this review (see Table 2). The included articles were predominantly from the USA (*n* = 18), with some studies conducted in Australia (*n* = 6) and the UK (*n* = 4). The remainder of the studies were from Germany (*n* = 2), Iran (*n* = 2), Singapore (*n* = 2), Turkey (*n* = 2) and 1 each from Denmark, China, Pakistan, Taiwan and Vietnam. A data summary table of the 41 articles can be found in Appendix B.

### 5.2. Demographics

Most studies included participants aged over 50 years, with a mean age of 71.19 years from 35 studies. In qualitative studies, the age range of participants of people who had fallen in hospital ranged from 17 to 92 years. People with cognitive impairment were excluded from most studies, with only five studies including those with mild cognitive impairment [23,24,25,26,27] and three studies including adults with mild to moderate cognitive impairment [28,29,30]. The study locations varied within the hospital and included emergency departments [24,31,32,33,34], sub-acute/rehabilitation settings [27,28,29,30,35], orthopaedic units [26,36,37], oncology units [38,39] and a bone marrow transplantation unit [40], with the remainder occurring in acute care wards. Of these, four studies took place across a range of wards [41,42,43,44], and one study occurred in a seven-hospital multi-site study across multiple wards [45].

### 5.3. Description of Fall Risk Perception Measures

There were variations in the constructs used to describe patients’ fall perceptions, with a total of 25 validated tools utilised to quantify fall perception. The single-item question “*are you afraid of falling?*” was the most frequently used fall perception measure [23,26,34,36,44,45,46], followed by the 16-item Fall Efficacy Scale-International (FES-I) [23,35,44,47,48,49] and the 7-item shortened version of the Fall Efficacy Scale-International [28,36,37,49,50]. The Falls Efficacy Scale (FES) also featured in five studies [24,48,49,51,52], with one study utilising the shortened FES [31]. Physiological fall risk tools were incorporated into some studies (*n* = 8) to compare patients’ perceptions with their actual fall risk. The Self-Awareness of Falls Risk Measure (SAFRM) was noted to be the only validated measurement tool that incorporated both the patients’ and clinicians’ perceptions of fall risk using the same measure [29,30].

Many studies utilised fall perception measures such as the FES and FES-I to measure fear of falling; however, it has been established that fear of falling and fall self-efficacy are different constructs [36]. In one study, participants’ self-efficacy improved after a fall question-and-answer education intervention [38], whereas there was a lack of significant findings on fall self-efficacy with the implementation of a multimedia fall prevention program [51]. Further, there were reports of an association between high medication use and lower fall self-efficacy and engagement in fall prevention strategies [51]. A low fall self-efficacy rating was also related to poor physical performance [36].

### 5.4. Description of Patients’ Perceptions of Fall Risk

A prominent emergent theme was the disparity between patients’ perceived fall risk and their clinical risk of falling. Patients did not consider themselves to be at risk of falling [32,39,40,46,53,54,55,56,57], and in three studies, approximately one-third of participants accurately identified their fall risk [28,29,44]. These statistics contrast with Radecki, Reynolds [58], as more than half of participants accepted that they were at risk of falling. Similarly, the findings of Greenberg, Moore [31] demonstrate alignment between participants’ perceived and actual risk. However, the tool used was not a validated fall risk assessment (Vulnerable Elders Survey). The importance of conducting comprehensive assessments was highlighted in Byrd [59]. In this study, clinicians were unaware of the presence of anosognosia in stroke participants, suggesting that these participants may have had inadequate fall prevention management. Despite fall prevention education, some patients overestimated their own ability in a hospital setting and were unaware that their fall risk could change with their medical condition [57]. A falls expert who recounted their own personal patient experience affirmed, “*Despite all the cues that nursing staff were giving me, I could not grasp that I was at high falls risk*” [60]. Evaluating both patients’ perceived and actual fall risk is essential to inform fall prevention education and strategies [44,55,61].

### 5.5. Description of Patients’ Perceptions of Falling in Hospital

The perception of the loss of independence and autonomy was highlighted in Gettens, Fulbrook [42] and Radecki, Reynolds [58], in which participants’ described their desire to be perceived as physically competent by others. Feelings of disappointment and disempowerment were expressed over their loss of independence after a hospital fall; however, this produced a behavioural change in which patients were more receptive to assistance [42]. These changes were also noticed in Turner, Jones [27], where participants reported increased reliance on nursing staff and a subtle shift in the locus of control after their falls. Self-blame with admissions of guilt over risk-taking behaviour was identified in Lim, Ang [57], with one person disclosing, “*It was because I refused to listen to other people’s advice. I wanted to take the risk to try* (walking) *by myself.*” An older adult’s motivation for maintaining independence and assuming risk-taking behaviours can be attributed to a desire to go home [43].

An emerging theme was patients’ lack of awareness over the causes of their hospital falls. Differing opinions were observed between patients and nurses in the work by Hoke and Zekany [54], in which patients attributed their falls to environmental factors, whereas nursing staff attributed their falls to “*not calling for assistance*.” Patients were more likely to blame extrinsic factors for their falls and did not understand the multifactorial basis behind falls [32]. Similarly, falls were perceived to be mechanical in nature and were referred to as a “loss of balance”, rather than to medication use or pre-existing conditions [27]. Patients were more receptive to interventions from health professionals following their hospital falls [32,42].

### 5.6. Description of Patients’ Fear of Falling

There were varied emotions and beliefs around the possibility of falling in hospital. Emotions ranged from apathy or no concern to extremely worried [32,62]. Falls were not considered to be a medical or life-threatening issue for some patients [57]; thus, some participants failed to see the consequences of a fall. The term “fear of falling” was frequently used in studies to determine patients’ fall perceptions and is associated with a range of adverse health and psychosocial outcomes [63]. A fear of falling was associated with higher levels of anxiety and reduced social support [26], reduced self-related quality of life scores and higher risk of falling [24] and higher dependency in activities of daily living (ADLs) [24]. There was also a higher association between fear of falling in women and those without a spouse [26,47,52]. Fear of falling increased after a hospital fall, with a reduction in confidence and reduced self-efficacy [27]. Self-perceived factors for increased fear of falling included balance difficulties, dyspnoea, muscle weakness and a history of falling [23,63].

### 5.7. Description of Barriers to Fall Prevention in Hospital

Patients’ thoughts and feelings about their own recovery were identified as the main barrier to engaging with fall prevention strategies [25]. Participants were more likely to engage in fall prevention if they viewed their fall risk as temporary rather than permanent [61]. In Twibell, Siela [56], 10% of participants acknowledged that they had no intention of using the call bell to request assistance when mobilising. Self-identity was important for participants, especially if they considered themselves to be strong and independent. Some participants had difficulty accepting fall prevention strategies that threatened their perceived self-identity, such as the use of a walking frame to ambulate [61].

Participants reported high confidence in the ability of the nursing staff to keep them safe. In Sonnad, Mascioli [46], 40% of patients did not consider themselves to be a fall risk because of high-quality nursing. Despite fall education delivered by nurses, the reduced use of the hospital call bell for requesting assistance was noted in some studies [50,54,62]. A common reason identified for this was that participants considered the nurses to be busy and did not want to impose on them [25,43,57]. Negative experiences or attitudes towards “unfriendly” nursing staff were also recognised as a factor in noncompliance with call bell use [43,57,62]. Some participants identified that delayed assistance from nurses instigated their risk-taking behaviour, leading to a risk of falling [25,43,58,62]. Valuing one’s dignity was considered a priority over potential falls. Avoiding incontinence and subsequent feelings of embarrassment took precedence over the risk of falling, as expressed by some participants [25,43,57,58,62].

## 6. Discussion

This scoping review explored the literature relating to patients’ perceptions of their fall risk in a hospital setting and their experiences of falling. To the best of our knowledge, this review is the first of its kind to investigate the scope of evidence around fall risk perceptions. Of the articles, 83% originated from high-income countries, as defined by the World Bank Group [64], with only 17% of studies conducted in low- to middle-income countries that met the inclusion criteria. As the majority of fall-related deaths occur in low- and middle-income countries [65], increased fall prevention efforts in low- and middle-income countries are essential.

Guidelines recommend that people over the age of 65 years be considered at risk of falling in hospital [66]. Interestingly, the studies that explored patients’ experiences of falling in hospital encompassed a wide age range, which suggests that all adult hospital inpatients could be considered at risk of falling. Fall risk assessment tools are traditionally completed by clinical staff to identify risk factors, thus producing an overall fall risk score in which individual interventions are implemented. This suggests that it is important to consider all hospital inpatients as a possible fall risk and to tailor fall prevention strategies accordingly. Fall risk assessment tools are traditionally completed by clinical staff to identify risk factors, thus producing an overall fall risk score in which individual interventions are implemented. Studies that divested from fall risk screening tools in favour of clinical reasoning reported “non-inferior” fall outcomes and potential improvements in fall rates [67,68]. Similarly, updated UK guidelines state: “*Do not use fall risk prediction tools to predict inpatients’ risk of falling in hospital*” [66]. The use of fall risk assessment tools can lead to complacency or a “checklist exercise”, resulting in inadequate fall prevention management. This highlights the importance of performing comprehensive multifactorial assessments and tailoring fall prevention strategies to the patient, rather than adopting a fixed approach.

A major finding from this scoping review, in line with the first research objective, is the disparity between patients’ fall perceptions and their physiological fall risk in hospital. This also confirms the qualitative findings of Heng, Slade [6] and of Dolan, Slebodnik [69], in which participants were not aware of their risk of falling despite having multiple risk factors for falling. Although this mismatch of fall risk is established, only one instrument (Self-Awareness of Falls Risk Measure) directly measures the fall risk disparity from the validated fall perception measures. The Self-Awareness of Falls Risk Measure is the first scale of its kind to measure self-awareness of fall risk in hospital and to quantify the disparities between clinicians’ and patients’ perceptions [70]. Under- or overestimations of fall risk are different constructs, meaning that the causes of these perceptions are varied, and management plans are dependent on their classification [70]. For example, a person who overestimates their fall risk will likely benefit from interventions geared towards their “fear of falling”, as opposed to someone who underestimates their fall risk and may otherwise engage in risk-taking behaviour. This approach to fall risk assessment aligns with current guidelines that recommend assessing the older person’s perceived functional ability and fear of falling [66]. The Self-Awareness of Falls Risk Measure may be of valuable use in a clinical setting, especially because it is also validated for those with mild to moderate cognitive impairment [70].

People with cognitive impairment are often excluded from gerontological research [71] yet have a higher risk of falling compared to those who are cognitively intact [72]. The term *anosognosia* is frequently associated with neurological impairments, in which patients are not aware of their physical deficits [73]. Anosognosia may be an important factor in explaining the discrepancy between actual and perceived fall risk in people with dementia, leading to risk-taking behaviour [63]. In one study, clinicians were unaware of the presence of anosognosia in 100% of the cases, potentially leading to inadequate fall management [59]. These findings demonstrate the importance of incorporating fall risk perception measures into assessments, especially for people with cognitive impairment [28].

Another prominent theme from the literature is the importance of patient dignity and perceptions of autonomy, which may influence compliance with a fall management plan. Feelings of disempowerment, loss of independence [42,58] and threats to perceived self-identity [61] demonstrate the vulnerability that older adults can experience in hospital. Basic human needs and personal care were fundamental to participants and were regarded as higher priorities than the possibility of falling [25,43,57,58,62]. Person-centred care involves seeking out and understanding what is important to the patient and adopting a collaborative approach based on elements such as respect, emotional support and care co-ordination [74]. Shared decision-making should feature in all healthcare settings as a pathway for health professionals and patients to work together to make decisions about care [75]. This verifies the importance of seeking patients’ perceptions and viewing subjective data as a valuable source of information to inform care and management [27].

Communication breakdown was identified as the overarching main barrier to patient engagement with fall prevention strategies. Whether it be decreased call bell use [43,50,54,56,62], prior negative experiences with nursing staff [43,57,62] or delayed assistance [25,43,58,62], communication failure could be attributed to various instances of noncompliance by patients. This also extended to interprofessional miscommunication between disciplines and on nursing clinical handovers [43]. To address communication issues, standardised communication tools have been devised, such as the SBAR tool (situation, background, assessment and recommendation) for interprofessional communication [76] or the TOP 5 intervention, which is five personalised important tips to aid communication between health professionals and people with dementia [77]. Evidence suggests that improving communication, partnering with patients and/or their families and seeking feedback lead to greater patient satisfaction and improved health and safety outcomes [74].

This review also determined that there were inconsistencies with patients’ perceptions of their causes of falling in hospital. Older adults were more likely to blame their falls in hospitals on external factors [27,32,54] and were unaware of contributing intrinsic factors, such as medication use or changes in medical conditions. These findings are comparable to Heng, Slade [6], who additionally identified that participants may have feelings of indifference towards fall education, as they did not consider it to be relevant to their needs. Patient fall education forms a considerable part of multifactorial fall interventions, in which guidelines recommend that individuals at risk of falling should be offered education orally and in writing [66]. Interestingly, a Cochrane review reported that the provision of educational materials may not affect the risk of falling in hospital, and there was very low-quality evidence of the effects of educational sessions on fall rates [3]. A meta-analysis has since found that education has a positive effect on hospital falls rate and risks, however further research is needed to determine optimal design and delivery [78]. The design and delivery of fall education should be individually tailored to the person, specific to their fall risk, and incorporate an active learning design for improved engagement [79].

“Fear of falling” or post-fall syndrome [80] describes people who have an anxiety of falling, which impacts their activity levels and independence, but may not have necessarily experienced a fall [81,82]. It is important to assess a person’s fear of falling along with their fall history to determine if they have a diagnosis of fear of falling syndrome [33]. Interestingly, Eckert, Kampe [36] found that fear of falling and fall self-efficacy are two separate constructs, yet some studies continue to incorporate self-efficacy measures to assess fear of falling. The Falls Efficacy Scale was developed based on the following definition: “*low perceived self-efficacy at avoiding falls during essential, nonhazardous activities of daily living*” [83]. Fall efficacy and confidence measures may not convey a true indication of fear of falling, as older adults may feel confident in activity engagement but may still harbour fears of potential falls [84]. This review exposes a gap and confirms that many studies continue to utilise the Falls Efficacy Scale measure and its variants to measure fear of falling in older adults. Given that these outcomes may not provide a true depiction of this phenomenon, further research should investigate these fall perception measures and their use within the clinical setting.

## 7. Implications for Future Research

In line with the second research objective, future research should focus on conducting a systematic review of existing fall risk perception measures to determine their suitability for use in a hospital setting. A comprehensive summary of their measurement properties and feasibility could be further investigated. In addition, researchers should consider the inclusion of people with cognitive impairment for future studies on fall perception, as their contribution should be valued.

## 8. Limitations

The limitations of this review include the use of English-language papers only. Scoping reviews are not intended to be a definitive synthesis of the literature; however, they are useful for disseminating research findings on a topic and identifying gaps in the literature [13,85]. Irrespective of these limitations, this review provides a valuable contribution to fall research by scoping the literature relating to patient perceptions of their fall risk in hospital.

## 9. Conclusions

This scoping review provides a detailed review of the research findings pertaining to patient perceptions of their fall risk in hospital. Approximately two-thirds of study participants did not accurately identify their fall risk compared to that defined by a health professional. This demonstrates the importance of partnering with patients to gain insight into their past experiences that may contribute to risk-taking behaviours. Regular collaboration with patients and seeking their feedback are also essential to communicating for safety. Opportunities for further research were identified in this review, which may provide meaningful contributions to improve fall knowledge on a global scale.

## Figures and Tables

**Table 1:** Keywords and search terms used.

Search Term	Variation
Fall *	Risk of falls
	Risk of falling
	Fall risk
Hospital	Ward
	Acute setting
	Emergency department
	Inpatient
Perception	Attitude
	Perspective
	Opinion
	View
	Experience
	Understanding
	Insight
	Self-awareness
	Awareness
	Fear of falling
	Anosognosia
	Ptophobia
NOT community	Community-dwelling
	Home
	Residential care
	Aged care
NOT paediatric	Pediatric
	Children

Key: * = truncated search term.

**Table 2:** Article characteristics.

Types of Studies	No. of Studies
Systematic literature reviews	2
Narrative reviews	1
Mixed methods studies	2
Qualitative studies	11
Randomised controlled trials	2
Quasi-experimental studies	1
Cross-sectional studies	7
Correlational studies	8
Cohort studies	2
Doctoral dissertations	3
Case reports	2
Total	41

## Data Availability

Data sharing is not applicable as no new data was created for this study.

## Appendix A. SR Protocol V5

Look on https://federationuniversity-my.sharepoint.com/personal/e_dabkowski_federation_edu_au/Documents/Desktop/Falls%20prevention/Writing/Publication/SR%20protocol%20V5.docx?web=1&wdLOR=c47B89699-58C4-41C9-9871-15B91A35D176 (accessed on 6 May 2022).

## Appendix B

**Table A1:** Data summary table.

Author, Year and Country	Study Aim	Study Design	Population	Fall Risk Perception Outcome Measures	Main Findings	Quality Appraisal
Beh et al. (2019), Ireland *“Older inpatients’ experience and insights into fear of falling: A feasibility study”*	To evaluate the effects of acute hospitalisation on fear of falling (FoF) among older adults and to evaluate older adults’ perceptions of risk factors, interventions and coping strategies for FoF	Mixed methods, single-item question and the FES-I. Patients with FOF completed a questionnaire. Descriptive statistics; qualitative data were presented as frequencies.	32 older inpatients aged > 65 years Mean age (SD): 75 (5)	Single-item question (SIQ), “are you afraid of falling?” Fall Efficacy Scale International (FES-I)	FoF did not appear to develop or change during hospitalisation. Self-perceived factors for increased FOF during hospitalisation were balance problems, breathlessness, muscle weakness and a history of falls. FoF was measured at a single time point during hospitalisation after hospital admission, or FoF on admission was compared to FoF after discharge. Patients perceived education and exercise prescription to be effective treatments for FoF post-hospitalisation. Patients who had significant cognitive impairment, were at the end of their life, were immobile, were critically ill or had acute psychiatric illnesses were excluded from the study.	57% (MMAT)
Byrd (2021), USA “*The relationship between anosognosia for hemiplegia after stroke and fall events in the acute inpatient stroke rehabilitation population”*	To explore the association between the presence of anosognosia for hemiplegia after stroke and patient fall events	Doctoral dissertation Prospective correlational study	16 ischaemic stroke patients in acute inpatient rehabilitation Mean age (SD): 57 (15.1)	Not specific to falls: Visual Analogue Test for Assessing Anosognosia for motor impairment	93.8% of stroke patients had a discrepancy score suggestive of anosognosia for hemiplegia (AHP). Clinicians at the bedside were unaware of the extent of the participants’ lack of awareness, indicating that the participant is at greater risk of falls. Limitations include small sample size and inclusion of ischaemic stroke patients only.	Thesis
Cerilo (2016), USA “*Effectiveness of fall prevention multimedia program on patient awareness, self-efficacy and engagement”*	To examine the effects of a multimedia program on hospitalised adults’ levels of fall risk awareness, self-efficacy and engagement in fall prevention	Doctoral dissertation Quasi- experimental study	60 inpatients in acute care aged >65 years Age range: 65 to 90	Falls Risk Awareness Questionnaire: 22 items Falls Efficacy Scale (FES)	There was a lack of significant findings on fall self-efficacy and engagement in fall prevention with the implementation of multimedia programs. Hospitalised adults who had high levels of fall prevention self-efficacy were more engaged in fall prevention efforts. The higher the number of medications that older adults were taking, the lower their levels of fall self-efficacy and engagement.	Thesis
Çinarli and Koç (2017), Turkey “*Fear and risk of falling, activities of daily living and quality of life*”	To describe risk and FoF in older adults seeking care in the ED and to explore relationships between risk and FoF with activities of daily living and quality of life	Cross-sectional study Structured questionnaires	151 older adults aged >65 years Mean age (SD): 72.7 (6.25)	FES	Patients with FoF showed a higher dependency in their activities of daily living (ADLs) and poorer self-related quality of life scores. There was a positive correlation between fear of falling and fall risk. ED offers an opportunity to assess fall risk and fear of falling to provide guidance on fall prevention and management.	50% (CASP)
Cox and Vassallo (2015), UK “*Fear of falling assessments in older people with dementia”*	To outline the key issues in relation to FoF, current guidelines and assessment tools and their use for people with dementia	Systematic literature review	4 studies	FES, FES-I, Icon FES	Further research is needed to address the assessment barriers that people with dementia may face regarding FoF tools. Self-reported questionnaires may be difficult for people with dementia to complete due to comprehension difficulties. Research in this area has predominantly been cross-sectional, with numerous factors being associated with FoF, including falls, co-morbidities, anxiety, polypharmacy and functional decline.	75% (SANRA)
Dadgari et al. (2020), Iran “*The relationship between the risk of falling and fear of falling among aged hospitalized patients”*	To determine the relationship between falling and fear of falling among aged hospitalised patients	Descriptive correlational study Questionnaires	385 hospitalised patients aged >60 years Mean age (SD): 71.68 (9.32)	FES	The evaluation of fear of falling and the risk of falls among hospitalised patients is recommended to predict the risk of falls. There was a statistical significance between FoF and women. There was also an association between FoF and those without a spouse.	58% (CASP)
Eckert et al. (2020), Germany “*Correlates of fear of falling and falls efficacy in geriatric patients recovering from hip/pelvic fracture*”	To gain a better understanding about the nature of fear of falling by analysing associations between psychological and physical aspects related to fear of falling and fall efficacy in hip/pelvic fracture patients	Baseline data of a randomised controlled trial (RCT) No interventions: cross-sectional data analyses completed	115 inpatients with hip/pelvic fracture aged >60 years Mean age (SD): 82.5 (6.8)	Short Fall Efficacy Scale International (Short FES-I), Perceived Ability to Manage Falls, SIQ FoF, Fall-related post-traumatic stress symptoms	Low fall efficacy was significantly related to poor physical performance. Low perceived ability to manage falls was significantly related to previous falls, psychological inflexibility and the female gender. FoF was directly associated with fall-related post-traumatic stress symptoms. The results confirm that fall efficacy and FOF are different constructs.	83% (CASP)
Gettens and Fulbrook (2015), Australia “*Fear of falling: association between the Modified Falls Efficacy scale, in-hospital falls and hospital length of stay”*	To investigate the relationships between fear of falling, fall risk, in-hospital falls and hospital length of stay	Observational non-experimental Design Descriptive and inferential statistics	141 inpatients (age range 17 to 95 years) Mean age (SD): 73.6 (15.5)	Modified FES (MFES)	Nearly all patients who fell had low MFES scores, which were associated with increased hospital length of stay (LOS). The lower the MFES scores, the higher their fall risk, indicating that it is a useful tool to predict in-hospital falls and increased hospital LOS.	75% (CASP)
Gettens et al. (2018), Australia “*The patients’ perspective of sustaining a fall in hospital: A qualitative study”*	To understand the patient’s perspective of falling in hospital	Qualitative -phenomenological Unstructured individualised interviews	12 hospital inpatients who had recently fallen (27 to 84 years) Mean age: 66	Thematic analysis: Van Manen’s approach	Three key themes emerged: feeling safe, realising the risk and recovering independence and identity. Participants had confidence in the nursing staff to keep them safe. Feeling disempowered and disappointed with their loss of independence but more receptive to receiving help. Some participants felt their autonomy was taken away Participants wanted others to perceive them as physically competent and thus were more likely to take risks.	90% (CASP)
Ghaffari- Rafi et al. (2019), USA “*Case report on fear of falling syndrome: A debilitating but curable gait disorder”*	A case study on a patient with fear of falling syndrome	Case report	70-year-old male presenting to ED	Case report: FES-I	This study highlights the importance of assessing fall history and FoF in older adults. The FES-I was used to assist in the diagnosis, as well as FoF gait (crouched posture, broader base and short stride length). The patient made a full recovery with anti-depressants, cognitive behavioural therapy and education.	75% (JBI)
Greenberg et al. (2016), USA “*Perceived fall risk and functional decline: Gender differences in patient’s willingness to discuss fall risk, fall history, or to have a home safety evaluation*”	To determine patient perceptions about their perceived fall risk compared to their actual risk of functional decline and death	Pilot prospective Study Descriptive and inferential statistics	146 adults presenting to ED aged >50 years Mean age (SD): 69 (11.4)	Shortened FES	There is an association between subjects’ perceived risk of falling and their risk of functional decline and death. This study used the FES as an indicator for perceived risk and the VES-13 for actual fall risk. The VES-13 is a tool used to identify older adults at risk of health deterioration.	50% (CASP)
Haines et al. (2012), Australia “*Why do hospitalized older adults take risks that may lead to falls?”*	To understand why older adults take risks that may lead to falls in the hospital setting and in the transition period following discharge home	Qualitative, phenomenological constructivist approach Semi-structured in-depth interviews and focus groups	Hospital patients aged >65 years (*n* = 16) Informal caregivers (*n* = 8) Health professionals (*n* = 33) Mean age (SD) of older adults: 75.4 (6.9)	Framework analysis	Five key factors that influence risk-taking behaviour were risk compensation ability of the older adult, willingness to ask for help, older adult desire to test their physical boundaries, communication failure and delayed provision of help. The challenge is to ensure that risk-taking behaviour by the older adult is informed and voluntary and undertaken in a supported environment.	100% (CASP)
Hauer et al. (2020), Germany “*Mismatch of subjective and objective risk of falling in patients with dementia”*	To analyse the mismatch between objective and subjective fall risk and associated factors in people with dementia	Cohort study Short FES-I, mobility assessments, functional assessments and psychological and fall-related behavioural strategies	173 inpatients with mild to moderate dementia Mean age (SD): 83.60 (6.16)	Short FES-I	Most patients demonstrated a mismatch between objective and subjective fall risk, with one-third of participants accurately identifying their fall risk. High levels of perceived fall risk are likely to result in a higher rate of falls, independent of physiological risk. The disparity between physiological and perceived fall risk is associated mainly with psychological and behavioural pathways.	75% (CASP)
Hill et al. (2016), Australia “*My independent streak may get in the way: how older adults respond to falls prevention education in hospital”*	To determine how providing individualised fall prevention education facilitated behaviour change from the perspective of older hospital patients on rehabilitation wards and what barriers they identified to engaging in preventive strategies	Prospective Qualitative Semi-structured questionnaire	Older patients (*n* = 610) aged >60 years, cognitively intact Mean age (SD): 81.4 (9.3)	Deductive content analysis	Participants thoughts and feelings about their recovery were the main barriers that they identified to engaging in safe strategies, including feeling overconfident or desiring to be independent and thinking that staff would be delayed in providing assistance. Educators reported that participants’ beliefs and attitudes were key influences in either facilitating or forming a barrier to engaging in fall prevention strategies.	90% (CASP)
Hoke and Zekany (2020), USA “*Two sides to every fall: Patient and nurse perspectives”*	To describe and categorise patient and nurse perspectives on falls and nurses’ suggestions for preventing falls	Qualitative descriptive study Individual bedside interviews providing narrative responses	67 patient falls (age range from 22 to 88 years) Mean age: 61	Content analysis	Three main themes emerged for all falls: activity, co-ordination and environment. 18% of the falls were witnessed, leading to nurse–patient agreement on the cause of the fall. 82% of falls were unwitnessed, occasionally resulting in disagreement about the cause of the fall. There were no patient perception measures undertaken prior to the patient fall. Many patients did not call for assistance, which the authors suggested was due to the participants not perceiving themselves to be at risk of falling.	70% (CASP)
Huang et al. (2015), Taiwan “*The effectiveness of a participatory program on fall prevention in oncology patients”*	To explore the effect of a participatory program on patients’ knowledge and self-efficacy of fall prevention and fall incidence in an oncology ward	Quasi- experimental study Pre-test and post-test to assess fall knowledge and self-efficacy	68 oncology patients (age range 19 to 77 years) Mean age: 47.8	15-item subscale about patients’ self-efficacy of fall prevention	Oncology patients had a higher self-efficacy of fall prevention after an intervention, after which they displayed better knowledge and concern about falls. Three subscale items that showed improvement in fall self-efficacy included getting in/out of bed, getting up to sit on the bed and standing up/sitting on a chair. Before the intervention, the average fall self-efficacy score indicated a moderate level of concern for falling in hospital by oncology patients.	89% (JBI)
Kakhki et al. (2018), Iran “*Fear of falling and related factors among older adults with hypertension in Tehran, Iran”*	To evaluate the factors involved in FoF in the elderly population with hypertension in Tehran, Iran	Descriptive correlative study Descriptive and inferential statistics	301 adults aged >60 years admitted to hospital Mean age (SD): 68.62 (6.82)	FES-I (Persian) FES-I	Two-thirds of participants with hypertension had a low FoF, and only 1% of participants reported a severe level of FoF. No meaningful relationship was identified between FoF and diseases other than hypertension. FoF was higher in women, people with a history of falls and people who lived alone.	50% (CASP)
Kiyoshi-Teo et al. (2019), USA “*Older hospital inpatients’ fall risk factors, perceptions, and daily activities to prevent falling”*	To identify associations among patient fall risk factors, perceptions and daily activities to improve patient engagement with fall prevention among hospitalised older adults	Cross-sectional Study Validated questionnaires	67 hospitalised patients aged >65 years Mean age (SD): 73.1 (6.4)	Short FES: 7-item Falls Behavioural Scale- Inpatient (FaB-I)	The frequency of daily activities to prevent falling was positively associated with concern about falling and level of health activation. Recent fall experience resulted in participants valuing fall prevention and engaging in fall prevention behaviours, but they reported decreased confidence in their ability to prevent a fall. Patients were reluctant to use their call light for mobility or to talk about fall prevention.	83% (CASP)
Kiyoshi-Teo et al. (2020), USA “*Qualitative descriptions of patient perceptions about fall risks, prevention strategies and self-identity: Analysis of fall prevention Motivational Interviewing conversations”*	To understand how older adults respond to fall prevention and identify attributes that affect their responses to fall prevention	Qualitative Individual motivational interviewing	30 hospital inpatients aged >65 years Mean age (SD): 72.83 (6.0)	Content analysis	Perceptions of fall risks were mostly formed by their current health condition and past fall experience. Participants were more likely to engage in fall prevention if they viewed their fall risk as temporary. Participants that perceived their fall risk as permanent had difficulty accepting their fall risk. Participants expressed more resistance to adopting fall prevention strategies that required major adjustments. Understanding older adults’ perceptions about their fall risks, prevention strategies and whether they align with their self-identity is essential for effective engagement in fall prevention.	90% (CASP)
Knox (2018), USA “*Fall risk perceptions: A study of hospitalized patients with hematologic malignancies”*	To describe the patient perceptions of fall risk in people with haematologic malignancies and compare patient and nurse perceptions of fall risk	Mixed methods Descriptive statistics and narrative analyses	15 hospitalised participants Age range: 36 to 86 years	Open-ended interview format to examine self-report of patients’ perceptions of fall risk	Participants who reported feeling weak prior to hospitalisation perceived being a high fall risk. Almost half of the participants (*n* = 7) had fallen in the past six months prior to hospitalisation; however, they attributed these falls to environmental causes. Some participants reported limited activity because of fatigue and had had to use assistive devices. Recommendations include conversing with patients about their understanding of fall risk when providing fall prevention education.	71% (MMAT)
Kronborg et al. (2016), Denmark “*Physical activity in the acute ward following hip fracture surgery is associated with less fear of falling”*	To objectively measure the physical activity the first week after hip fracture surgery and relate it to functional performance and fear of falling at discharge	Observational Study Functional measures, accelerometer, questionnaires	38 older adults aged >65 years at a hip fracture unit Mean age (SD): 80 (8.4)	Short FES-I	In participants who underwent orthopaedic surgery, there was a positive association between more time spent upright, independent mobility and a decreased fear of falling one week after surgery.	75% (CASP)
Kuhlenschmidt et al. (2016), USA “*Tailoring education to perceived fall risk in hospitalized patients with cancer”*	To determine the effect of tailored, nurse-delivered interventions as compared to a control group on patient perception of risk of falls, confidence in fall prevention and willingness to ask for assistance	Randomised controlled design Provision of individualised education tailored to the nurse’s risk assessment and the patient’s perception of fall risk	91 participants with cancer (*n* = 47 in control group and *n* = 44 in intervention group) Mean age (SD): 58.79 (14.25)	Patients self-reported their perceived risk of falls, their confidence to prevent a fall and their willingness to ask for assistance	Oncology nurses should incorporate a structured evaluation of patient perception of their risk factors. There is a need for assessment tools and interventions to realign discrepancies in perceptions of fall risk between nurses and patients. The intervention of individualised education was not effective in changing willingness to call for assistance, as most people in the intervention group reported a high willingness to ask for assistance.	73% (CASP)
Lim, Ang, et al. (2018), Singapore “*Patients’ experience after a fall and their perceptions of fall prevention: A qualitative study*”	To explore the experiences of patients who had a fall and their perspectives toward fall prevention in the acute care setting	Qualitative– exploratory, descriptive study Individual interviews one day after hospital fall	100 patients Mean age (SD): 65.2 (12.1)	Inductive content analysis	Six main themes emerged: apathy towards falls, self-blame behaviour, reluctance to impose on busy nurses, negative feelings towards busy nurses, overestimating own ability and poor retention of information. Falls were not deemed as a medical event or life threatening; thus, many failed to see the potential consequences of a fall.	90% (CASP)
Lim, Seow, et al. (2018), Singapore “*Disparity between perceived and physiological risks of falling among older patients in an acute care hospital*”	To describe differences between perceived and actual physiological risk of falling among older adults and to explore factors associated with the differences	Prospective cohort study Perceived fall risk measures and physiological fall risk scale	300 inpatients (age >65 years) Mean age (SD): 75.3 (6.2)	Single item: “are you afraid of falling?” FES-I	Only one-third of patients accurately perceived their fall risk. Patients on laxatives were more likely to be aware of their fall risk. Both patients’ perceived and actual fall risks should be evaluated to inform individualised fall prevention education and strategies. Patients who had a fall in the six months prior to hospitalisation were more likely to be aware of their own fall risks.	83% (CASP)
Mihaljcic et al. (2015), Australia “*Self-awareness of falls risk among elderly patients: Characterizing awareness deficits and exploring associated factors”*	To characterise self-awareness in older adults undergoing inpatient rehabilitation and explore factors associated with reduced awareness of fall risk	Prospective, cross-sectional SAFRM, timed up and go test and cognition	91 older adults undergoing inpatient rehabilitation aged >60 years Mean age (SD): 77.97 (8.04)	Self-awareness of falls risk (SAFRM): 31 items	A significant number of older adults undergoing inpatient rehabilitation underestimated personal fall risk (59%). Neurologic history was associated with lower intellectual and overall self-awareness. Men demonstrated a trend towards lower levels of self-awareness than women.	83% (CASP)
Mihaljcic et al. (2017), Australia “*Investigating the relationship between reduced self-awareness of falls risk, rehabilitation engagement and falls in older adults”*	To investigate whether self-awareness of fall risk is associated with rehabilitation engagement, motivation for rehabilitation and number of falls after hospital discharge	Correlational study Questionnaires including SAFRM, rehabilitation engagement, motivation for rehabilitation, cognition and functional ability	91 inpatients in rehabilitation (age range: 62 to 93 years) Mean age (SD): 77.97 (8.04)	Self-awareness of falls risk (SAFRM): 31 items	Reduced self-awareness is associated with lower self-reported motivation for rehabilitation and lower clinician-reported engagement in rehabilitation. Self-awareness demonstrated the strongest association with occupational therapy-rated engagement. Intellectual and anticipatory awareness demonstrated significant correlations with engagement in physiotherapy.	83% (CASP)
Mion (2016), USA “*When the falls expert becomes the fall risk patient: Through the looking-glass”*	Personal experience as a fall risk patient	Case report	Author’s personal experience Age: 60	Personal experience	The author did not understand that she was at risk of falling despite all of the cues and fall prevention strategies from the nursing staff. If the patient does not grasp the concept of being at risk of falls, patient education and patient reminders may not work. Continuous reinforcement of fall prevention strategies may be worthwhile.	88% (JBI)
Nguyen et Al. (2020), Vietnam “*Fear of falling among older patients admitted to hospital after falls in Vietnam: Prevalence, associated factors and correlation with impaired health-related quality of life”*	To examine the fear of falling in older patients hospitalised due to fall injuries, its effect on health-related quality of life and its associated factors	Secondary analysis from a multi-site cross-sectional study Structured questionnaire via face-to-face interviews	405 inpatients (aged >60 years) Mean age (SD): 71.9 (9.0)	Single close-ended Question: “Are you afraid of falling?”	88.2% of participants reported FoF after their falls in which their injuries required hospitalisation in Vietnam. Older people with psychological problems are more likely to report FoF, along with a history of eye disease. Other factors associated with FoF include living alone, use of mobility aids and living with children. FoF had an independent negative relationship with the HRQOL questionnaire.	75% (CASP)
Peeters et al. (2020), Ireland “*Understanding the aetiology of fear of falling from the perspective of a fear-avoidance model: A narrative review”*	To review the literature on physiological, mood and cognitive factors associated with fear of falling and to interpret these findings in the context of a fear- avoidance model that provides a causal framework for the development of FoF	Narrative review	52 studies	Data was synthesized on a narrative level to generate several hypotheses of the mechanisms explaining FoF	Fear of falling is associated with a range of adverse health and psychosocial outcomes. Evidence suggests that fear of falling is influenced by balance problems and falls and cognitive factors. Anosognosia or lack of awareness of disease or disability may be an important factor in explaining the discrepancy between actual and perceived fall risk in people with dementia. The authors proposed extending the fear avoidance model to include cognitive function, depression and neuroticism.	83% (SANRA)
Pena (2019), USA “*Patient perception of fall risk and high fall risk screening scores”*	To describe the relationship between patient perception of fall risk and high fall risk screening scores	Doctoral dissertation Descriptive correlational design	201 inpatients (aged >65 years) Mean age (SD): 77.1 (7.9)	Four scales: The fear of falling while hospitalised scale, the confidence to engage in fall prevention scale, the intention to engage in fall prevention scale and the consequences of falling while hospitalised scale	Older adults generally do not view themselves as at risk of falling. The participants in this study were not fearful they would fall, were confident they would not have a fall, intended to call for help when getting out of bed and had a neutral perception of enduring severe consequences if they did have a fall while in hospital. Incorporating patients’ perceptions into their care may improve patient engagement.	Thesis
Radecki et Al. (2018), USA “*Inpatient fall prevention from the patient’s perspective: A qualitative study”*	To describe the patient’s perspective of fall prevention in an acute care setting to aid in the design of patient-centred strategies	Qualitative Semi- structured interviews	12 inpatients (age range: 38 to 89 years) Mean age: 65.2	Thematic analysis	More than half of the patients considered themselves to be a fall risk due to their physical limitations. Some patients described the insecurity and vulnerability of being a fall risk and their lack of independence. The most frequently mentioned barrier was the time spent waiting for the nurse, in which their need for the bathroom overrode fall nurse instructions. More research is needed to develop an inpatient self-assessment tool that may help patients recognise their risk factors and become a more active and accepting participant in their fall prevention strategies.	90% (CASP)
Rizwan et al. (2020), Pakistan “*Fear of falling among sub-acute stroke patients in Lahore, Pakistan*”	To compare fear of fall (lack of self-confidence to maintain balance during normal activity) with and without fall history among sub-acute stroke patients in Lahore, Pakistan	Cross-sectional study	66 sub-acute stroke patients (age range: 40 to 75 years)	FES-I	There was a significant association between fear of fall and history of falls in stroke patients. FES-I scores were higher in stroke patients with a history of falls than in patients without a history of falls. Participants with neurological diseases other than stroke, lower extremity procedures or other surgeries during the past 6 months of the study were excluded.	58% (CASP)
Savas et al. (2019), Turkey “*Factors related to falls and the fear of falling in Turkish elderly patients admitted to emergency department”*	To investigate the fear of falling and admissions related to falls, as well as the factors associated with each of them, among elderly patients who are admitted to the ED	Cross-sectional Study Descriptive and inferential statistics	555 older adults presenting to ED aged >65 Years Mean age (SD): 76.7 (7.6)	SIQ: fear of falling	There was a significant relationship between falls and FoF among older patients admitted to ED. FoF was associated with living in a nursing home, past history of falls and independence in ADLs. 12.6% of participants were admitted because of falls.	67% (CASP)
Scholz et al. (2021), USA “*Fear of falling and falls in people with multiple sclerosis: A literature review”*	To provide an overview of existing research on the effects of FoF and therapy options in multiple sclerosis (MS)	Literature review	35 articles	FES-I, 7-item FES-I, 10-item FES, Survey of Activities and FoF in the Elderly, Spinal Cord Injury Fall Concerns Scale, Fear of Falling Avoidance Behaviour Questionnaire, Activities-specific Balance and Confidence Scale, University of Illinois at Chicago FoF measure	The FES-I was the most frequently used instrument to assess FoF for people with MS. People with higher FoF scores had an increased number of falls, lower walking speed, shorter stride length, larger sway and a more severe disability. FoF is multifactorial and includes motor and non-motor factors. Therapies should incorporate both physical and psychological aspects in neurorehabilitation. Most of the studies in this review were cross-sectional designs; thus, no causal associations between FoF, falls and disabilities can be assumed.	92% (SANRA)
Shankar et al. (2017), USA “*Exploring older adult ED fall patients’ understanding of their fall: A qualitative study”*	To understand older patients’ perspectives about their fall, fall risk factors and attitude towards emergency department fall- prevention interventions	Qualitative Semi-structured interviews	63 participants aged >65 years at the ED following a fall Mean age (SD): 79.9 (8.5)	Thematic analysis	Patients with some concern over future falls were able to name some modifiable risk factors. Patients with little to no concern of future falls minimised any risk factors or already partook in their own perceived risk-reducing activities. The reasons for patient falls were circumstantial and included environmental factors, accidental/carelessness or due to a specific medical condition. Older adult ED fall patients lacked understanding about their fall risk and had varied perceptions about their future fall risk.	80% (CASP)
Shuman et al. (2016), USA “*Patient perceptions and experiences with falls during hospitalization and after discharge*”	To describe hospitalised older adults’ (>60 years) perceptions about their fall risks while hospitalised, the fall prevention interventions received while hospitalised and their fall prevention discharge instructions	Qualitative– prospective, exploratory Two individualised semi-structured interviews per participant (during hospitalisation and via telephone post-discharge	15 patients (aged >60 years) 3 participants completed the first interview only Mean age (SD): 72 (10.86)	Constant comparative methods	Eight major themes emerged: overall perceptions of falling, overall perceptions of fall prevention interventions, “telling” fall prevention by hospital staff, “doing” fall prevention, effectiveness of fall prevention strategies, personal fall prevention strategies and fall-related discharge instructions. Most participants stated that they did not perceive themselves to be at risk of falling while in the hospital. Most participants had fallen prior to hospitalisation, and they were able to identify contributing factors. Nurses and healthcare providers should have multiple conversations with hospitalised patients and their families about why they are at risk of falling and define the specific risk factors they have that may contribute to a fall or injury from a fall.	90% (CASP)
Sonnad et al. (2014), USA “*Do patients accurately perceive their fall risk?”*	To document patient perceptions of their inpatient fall risk and determine how these perceptions were associated with clinical indicators of fall risk	Prospective survey-based design Survey and medical record to obtain Schmid score	92 inpatients Mean age: 60.77	Single close-ended question: “Are you afraid of falling?”	Patient perceptions of falls may not always match their clinical risk or actual likelihood of falling. Patients who perceived themselves as at risk of falling cited balance, injury, nausea, recent falls or concerns about the equipment they were connected to as their main issues. High-quality nursing may instil a false sense of security in patients, with 40% of patients reporting that they did not consider themselves to be at risk of falling due to the nursing support. More research may be required to understand why patients do not perceive themselves to be at risk of falling.	58% (CASP)
Turner et al. (2019), UK “*The perceptions and rehabilitation experience of older people after falling in the hospital”*	To explore the experiences of older patients who fell during their hospital stays	Qualitative– exploratory Semi-structured interviews, incident reports and medical records	5 inpatients (age range: 77 to 88 years) Mean age: 81.2	Thematic, discourse and descriptive analysis	Overarching themes include causes of falling, changes in mobility, changes in confidence, self-efficacy and attitude toward rehabilitation and the role of the staff. A loss of balance was reported to be the main reason why patients fell. Participants reported reduced confidence, low self-efficacy and less positive attitudes towards their rehabilitation following their fall.	100% (CASP)
Twibell et al. (2020), USA “*Perspectives of inpatients with cancer on engagement in fall prevention”*	To explore perspectives of hospitalised adults with cancer regarding engagement in fall prevention plans. The secondary aim was to compare fall-related perspectives of patients who had and who had not fallen	Qualitative– descriptive, exploratory Individual interviews at the bedside	30 inpatients with cancer (age range: 26 to 92 years) Mean age: 65.4	Thematic analysis	No participants reported an increased vulnerability to falling because of their cancer. The majority of participants did not believe that they would fall or that they would experience negative consequences if they fell. Delays in responses to calls for assistance discouraged participants from calling for help in the future. Participants expressed irritation about choosing between being incontinent if help did not arrive in time or being “in trouble” for disregarding the prevention plan if they independently mobilised.	100% (CASP)
Twibell et al. (2015), USA “*Perceptions related to falls and fall prevention among hospitalized adults*”	To explore hospitalised adults’ perceptions related to risk of falling, fear of falling, expectations of outcomes of falling and intention to engage in behaviours to prevent falls	Correlational study 4 questionnaires and 3 single-item surveys	158 acute inpatients (age range 31 to 98 years) Mean age (SD): 69.9 (13.37)	The Confidence to Perform Without Falling Scale, Fear of Falling While Hospitalised Scale, Consequences of Falling While Hospitalised Scale, Intention to Engage in Fall Prevention scale and 3 single-item surveys: perceived likelihood of falling while hospitalised, perceived likelihood of injury if they did fall while hospitalised and perceived fear of falling	Participants with a low intention to engage in fall prevention reported low fear of falling, low perceived likelihood of adverse outcomes from falling, few consequences of falling and high confidence in safely performing risky behaviours. Fear of falling is a key perception for nurses to assess in designing fall prevention plans. There is a mismatch between nurses’ and patients’ evaluations of patients’ risk of falling; more than half of the participants did not perceive that they were likely to fall. 10% of participants did not intend to call for assistance when performing any behaviour associated with risk of falling.	83% (CASP)
Zhang et al. (2021), China “*Incidence and risk factors related to fear of falling during the first mobilisation after total knee arthroplasty among older patients with knee osteoarthritis: A cross-sectional study”*	To examine the fear of falling among patients who underwent a TKA and to determine the factors that are associated with that fear	Cross-sectional study Validated questionnaires	285 inpatients aged >65 years who had a TKA Mean age (SD): 75.2 (6.4)	Single close-ended question: “In general, are you afraid of falling?”	Over half of the participants reported having FoF. FoF was more frequent in women, those living alone and participants with a higher BMI. FoF was also more frequent in participants with a higher level of anxiety or reduced social support. Future studies could include a qualitative component to explore the emotional and psychological dimensions of fear of falling among older patients.	83% (CASP)

**Abbreviations:** ADLs: activities of daily living; AHP: anosognosia for hemiplegia; CASP: Critical Appraisal Skills Programme; ED: emergency department; FaB-I: Falls Behavioural Scale-Inpatient; FES: Falls Efficacy Scale; FES-I: Falls Efficacy Scale-International; FoF: fear of falling; FRAT: fall risk assessment tool; HRQOL: health-related quality of life; JBI: Joanna Briggs Institute; LOS: length of stay; MFES: Modified Falls Efficacy Scale; MMAT: mixed methods appraisal tool; MS: multiple sclerosis; RCT: randomised controlled trial; SAFRM: self-awareness fall risk measure; SANRA: Scale for the Assessment of Narrative Review Articles; SD: standard deviation; SIQ: single-item question; TKA: total knee arthroplasty; VES: Vulnerable Elders Survey.
